# Supplementary material for: Neural codes track prior events in a narrative and predict subsequent memory for details
Source: Commun Psychol. 2025 Feb 16;3:26. doi: 10.1038/s44271-025-00211-y (PMC11830764; doi:10.1038/s44271-025-00211-y)
Supplement: Supplementary file 2 — Supplemental Information [file 44271_2025_211_MOESM2_ESM.pdf]

Supplementary Information

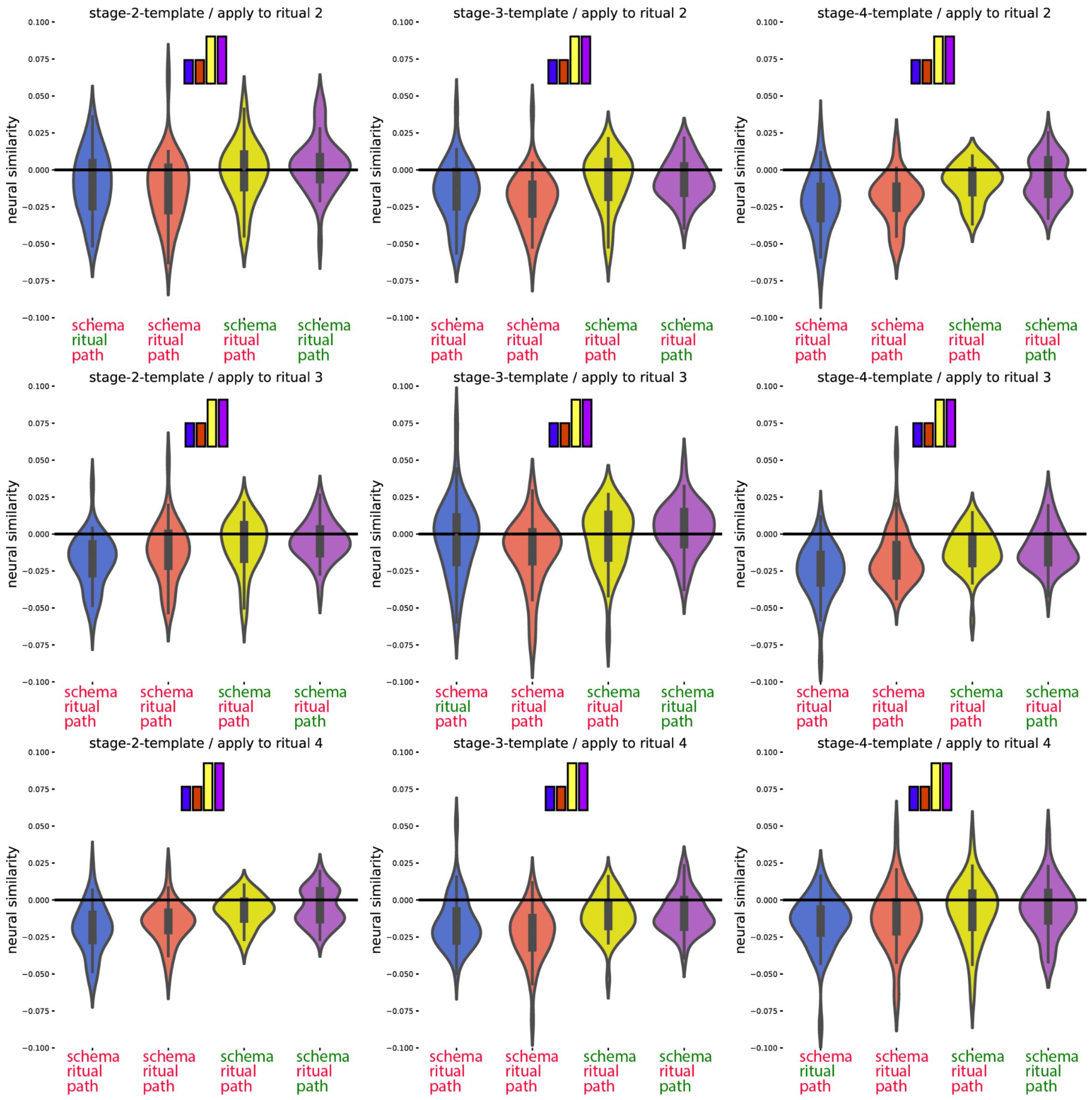

Supplementary Figure 1 Posthoc confirmation of the pattern for the regions identified in Figure 5 as showing the schema neural code (Figure 6 shows the same results, separately averaging across all within-stage and across-stage comparisons). Colors of violin plots indicate the relationship between the paths of the template and the held-out ritual: Purple = same path; yellow = different path, same schema; orange = different schema, always different ritual; blue = different schema, same ritual for within-stage comparisons (but not across-stage comparisons). For convenience, colors of x axis labels indicate whether the schema/ritual/path are the same (green) or different (red) for the template and held-out ritual. The 9 sub-figures correspond to different combinations of using stage 2/3/4 as the template and applying the template to stage 2/3/4. The small bars at the top of each plot indicate the predicted pattern.

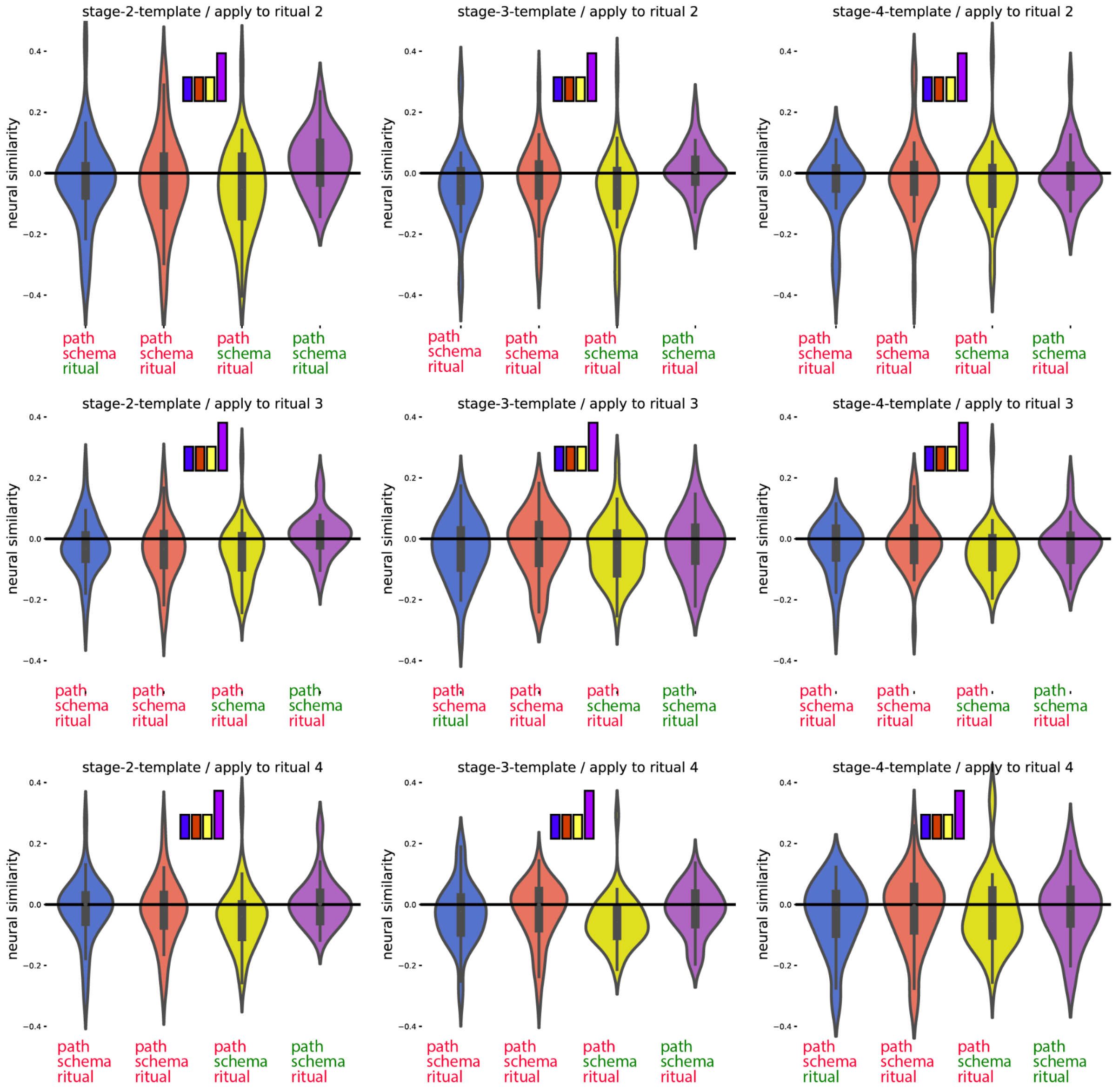

Supplementary Figure 2 Posthoc confirmation of the pattern for the regions identified in Figure 5 as showing the path neural code (Figure 7 shows the same results, separately averaging across all within-stage and across-stage comparisons). Colors of violin plots indicate the relationship between the paths of the template and the held-out ritual: Purple = same path; yellow = different path, same schema; orange = different schema, always different ritual; blue = different schema, same ritual for within-stage comparisons (but not across-stage comparisons). For convenience, colors of x axis labels indicate whether the schema/ritual/path are the same (green) or different (red) for the template and held-out ritual. The 9 sub-figures correspond to different combinations of using stage 2/3/4 as the template and applying the template to stage 2/3/4. The small bars at the top of each plot indicate the predicted pattern.

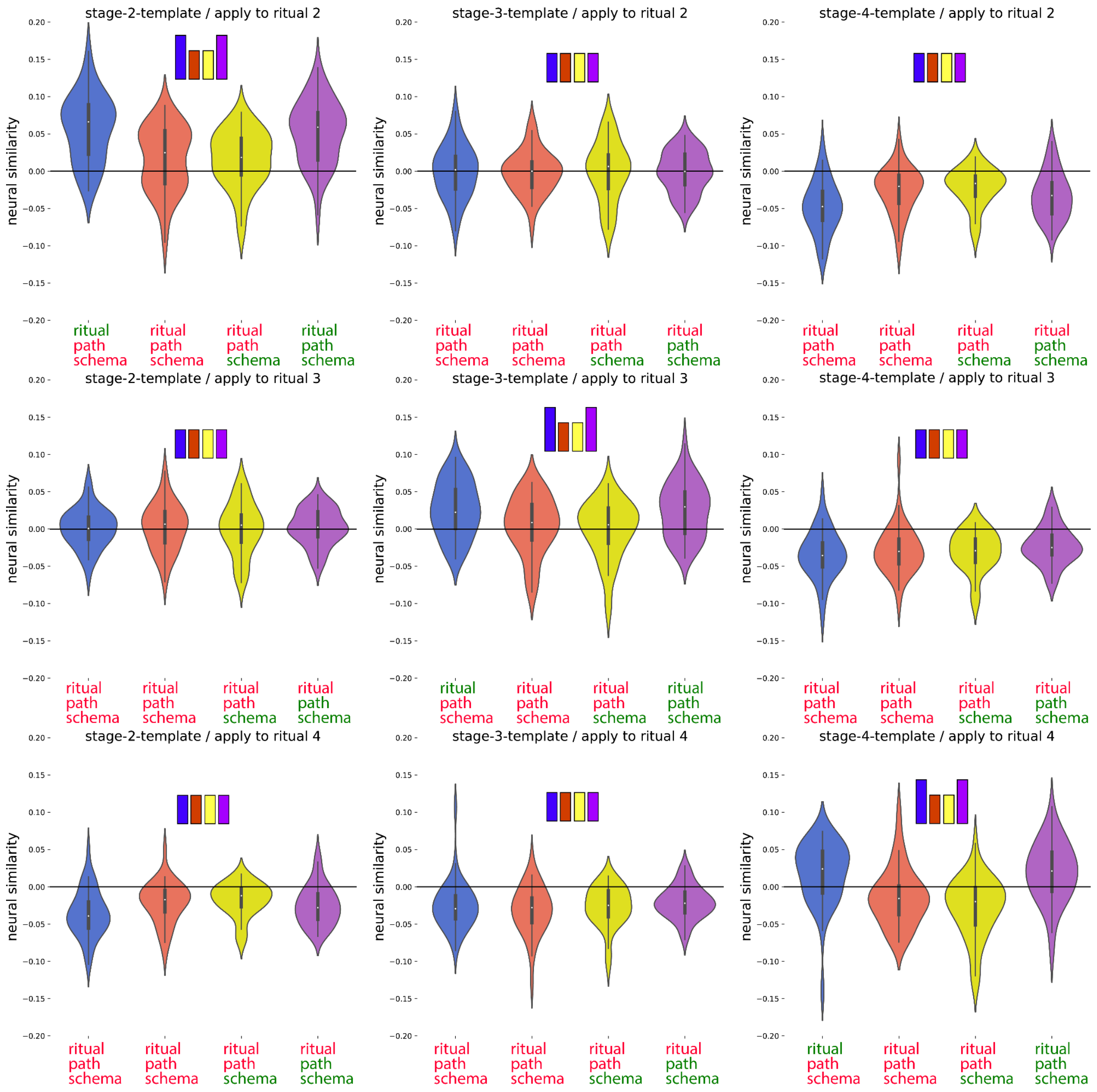

Supplementary Figure 3 Posthoc confirmation of the pattern for the regions identified in Figure 5 as showing the current ritual neural code but not the rotated preceding ritual neural code. (Figure 10 shows the same results, separately averaging across all within-stage and across-stage comparisons). Colors of violin plots indicate the relationship between the paths of the template and the held-out ritual: Purple = same path; yellow = different path, same schema; orange = different schema, always different ritual; blue = different schema, same ritual for within-stage comparisons (but not across-stage comparisons). For convenience, colors of x axis labels indicate whether the schema/ritual/path are the same (green) or different (red) for the template and held-out ritual. The 9 sub-figures correspond to different combinations of using stage 2/3/4 as the template and applying the template to stage 2/3/4. The small bars at the top of each plot indicate the predicted pattern.

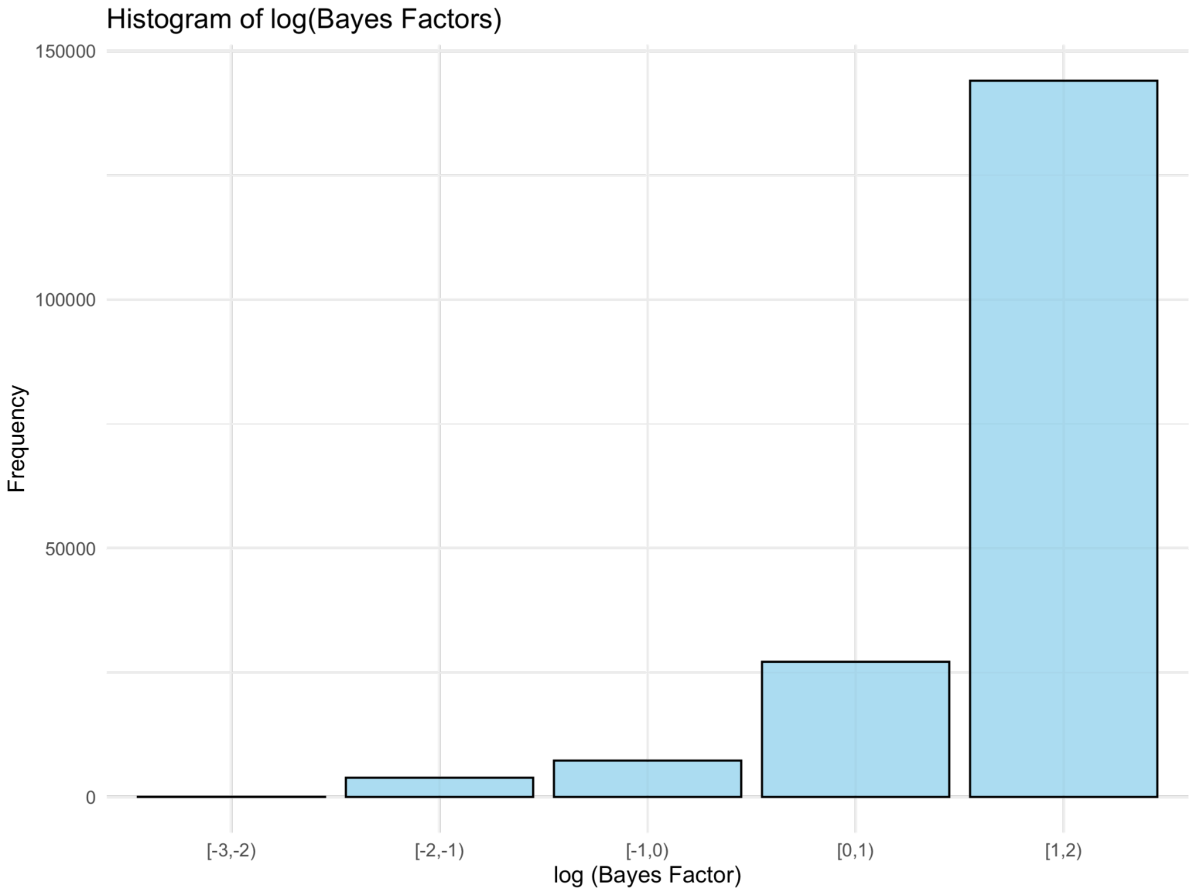

Supplementary Figure 4 A histogram of $\log_e$ Bayes Factors (BF01) across all searchlights of the whole brain for the non-rotated preceding ritual code analysis. Positive values indicate more evidence for the null hypothesis.

Supplementary Table 1: Full statistics (related to Figure 6).

**Within-stage subplot**					
**effect**	**t**	**p**	**ES**	**CI 95%**	**BF-01**
schema effect	4.214	< 0.001	d=0.666	0.320, 1.006	
within-schema path effect	1.230	0.226	d=0.195	-0.120, 0.506	2.911
current ritual effect	2.041	0.048	d=0.323	0.003, 0.639	
**Across-stage subplot**					
**effect**	**t**	**p**	**ES**	**CI 95%**	**BF-01**
schema effect	6.310	< 0.001	d=0.998	0.613, 1.374	
within-schema path effect	0.690	0.494	d=0.109	-0.202, 0.419	4.690

Supplementary Table 2: Full statistics (related to Figure 7).

**Within-stage subplot**					
**effect**		**p**	**ES**	**CI 95%**	**BF-01**
schema effect	W = 393	0.826	rb=- 0.041	-0.377, 0.304	5.197
within-schema path effect	t = 2.514	0.016	d=0.397	0.073, 0.717	
current ritual effect	W = 477	0.375	rb=0.163	-0.188, 0.478	2.445
**Across-stage subplot**					
**effect**		**p**	**ES**	**CI 95%**	**BF-01**
schema effect	W = 354	0.460	rb=-0.137	-0.457, 0.215	5.732
within-schema path effect	t = 3.109	0.003	d=0.492	0.160, 0.817	

Supplementary Table 3: Full statistics (related to Figure 8).

**Stage2 template-apply to ritual 2 subplot**					
**effect**	**t**	**p**	**ES**	**CI 95%**	**BF-01**
schema effect	-1.138	0.262	d=-0.180	-0.491, 0.134	3.214
within-schema path effect: confounded with current ritual effect, so not tested					
current ritual effect	9.383	< .001	d=1.484	1.028, 1.93	
same upcoming ritual effect	- 1.072	0.290	d = -0.169	-0.481, 0.144	3.438
**Stage3 template-apply to ritual 2 subplot**					
**effect**	**t**	**p**	**ES**	**CI 95%**	**BF-01**
schema effect	-0.015	0.988	d=-0.002	-0.312, 0.308	5.861
within-schema path effect: confounded with rotated preceding ritual effect, so not tested					
rotated preceding ritual effect	5.770	< .001	d=0.912	0.538, 1.278	
**Stage2 template-apply to ritual 3 subplot**					
**effect**	**t**	**p**	**ES**	**CI 95%**	**BF-01**
schema effect	0.294	0.771	d=0.046	-0.264, 0.356	5.629
within-schema path effect: confounded with rotated preceding ritual effect, so not tested					
rotated preceding ritual effect	2.698	.01	d=0.427	0.100, 0.748	
**Stage3 template-apply to ritual 3 subplot**					
**effect**	**t**	**p**	**ES**	**CI 95%**	**BF-01**
schema effect	-0.818	0.419	d=-0.129	-0.440, 0.183	4.289
current ritual effect	4.204	< .001	d=0.665	0.318, 1.004	

same preceding ritual effect	2.698	.01	d=0.427	0.100, 0.748	

Supplementary Table 4: Full statistics (related to Figure 9).

**Stage2 template-apply to ritual 2 subplot**					
**effect**	**W**	**p**	**ES**	**CI 95%**	**BF-01**
schema effect	576	0.025	rb=0.405	0.074, 0.656	
within-schema path effect: confounded with current ritual effect, so not tested					
current ritual effect	298	0.135	rb=-0.273	-0.562, 0.075	4.070
same upcoming ritual effect	621	0.004	rb = 0.515	0.210, 0.728	
**Stage3 template-apply to ritual 2 subplot**					
**effect**	**W**	**p**	**ES**	**CI 95%**	**BF-01**
schema effect	549	0.062	rb=0.339	-0.003,0.610	0.436
within-schema path effect: confounded with rotated preceding ritual effect, so not tested					
rotated preceding ritual effect	254	.036	rb=-0.380	-0.639, -0.04	
**Stage2 template-apply to ritual 3 subplot**					
**effect**	**W**	**p**	**ES**	**CI 95%**	**BF-01**
schema effect	560	0.044	rb=0.366	0.028, 0.629	
within-schema path effect: confounded with rotated preceding ritual effect, so not tested					
rotated preceding ritual effect	264	.05	rb=-0.356	-0.622, -0.017	
**Stage3 template-apply to ritual 3 subplot**					
**effect**	**W**	**p**	**ES**	**CI 95%**	**BF-01**

schema effect	549	0.062	rb=0.339	-0.003, 0.610	0.563
current ritual effect	575	0.026	rb=0.402	0.071, 0.654	
same preceding ritual effect	304	0.158	rb=-0.259	-0.551, 0.091	4.764

Supplementary Table 5: Full statistics (related to Figure 10).

**Within-stage subplot**					
**effect**	**t**	**p**	**ES**	**CI 95%**	**BF-01**
schema effect	-1.472	0.149	d=-0.233	-0.545, 0.083	2.169
within-schema path effect: confounded with current ritual effect, so not tested					
current ritual effect	8.796	< 0.001	d=1.391	0.950, 1.823	
**Across-stage subplot**					
**effect**	**t**	**p**	**ES**	**CI 95%**	**BF-01**
schema effect	1.888	0.066	d=0.299	-0.020, 0.614	1.173
within-schema path effect	-0.107	0.916	d=-0.017	-0.327, 0.293	5.830
